# Presentation to Sir George Buchanan

**Published:** 1892-06

**Authors:** 


### Presentation to Sir George Buchanan, F.R.S.

As Sir George Buchanan has lately resigned the post of Medical Officer of the Local Government Board, it is proposed to present him with some permanent memento of the esteem in which he and his work are held.

For the purpose of forwarding this movement, a strong Committee has been formed, of which Dr. Bristowe is Treasurer. Dr. Davies, the Medical Officer of Health for Bristol, who is a member of the Committee, will be glad to send to the proper quarter any contributions that may be entrusted to him.

